# TSP50 promotes hepatocyte proliferation and tumour formation by activating glucose‐6‐phosphate dehydrogenase (G6PD)

**DOI:** 10.1111/cpr.13015

**Published:** 2021-02-25

**Authors:** Xiaojun Zhang, Feng Gao, Huihan Ai, Shuyue Wang, Zhenbo Song, Lihua Zheng, Guannan Wang, Ying Sun, Yongli Bao

**Affiliations:** ^1^ National Engineering Laboratory for Druggable Gene and Protein Screening Northeast Normal University Changchun China; ^2^ Research Center of Agriculture and Medicine Gene Engineering of Ministry of Education Northeast Normal University Changchun China

**Keywords:** acetylation, G6PD, HCC, proliferation, TSP50

## Abstract

**Background & Aims:**

Hepatocellular carcinoma (HCC) is a common malignant tumour with high morbidity and mortality. Metabolic regulation by oncogenes is necessary for tumour growth. Testes‐specific protease 50 (TSP50) has been found to promote cell proliferation in multiple tumour types. However, the mechanism that TSP50 promotes HCC progression are not known.

**Methods:**

Hepatocyte proliferation was analysed by MTT and BrdU incorporation after TSP50 transfection. Furthermore, LC‐MS/MS, co‐immunoprecipitation and GST pull‐down assays were performed to analyse protein(s) binding to TSP50. Moreover, the site‐specific mutation of G6PD was used to reveal the key site critical for G6PD acetylation mediated by TSP50. Finally, the role of G6PD K171 acetylation regulated by TSP50 in cell proliferation and tumour formation was investigated.

**Results:**

Our data suggest that the overexpression of TSP50 accelerates hepatocyte proliferation. In addition, G6PD is an important protein that binds to TSP50 in the cytoplasm. TSP50 activates G6PD activity by inhibiting the acetylation of G6PD at the K171 site. In addition, TSP50 promotes the binding of G6PD to SIRT2. Furthermore, the K171ac of G6PD regulated by TSP50 is required for TSP50‐induced cell proliferation in vitro and tumour formation in vivo. Additionally, according to The Cancer Genome Atlas (TCGA) programme, TSP50 and G6PD are negatively correlated with the survival of HCC patients.

**Conclusions:**

Collectively, our findings demonstrate that TSP50‐induced cell proliferation and tumour formation are mediated by G6PD K171 acetylation.

## Graphical abstract

Zhang et al show that serine protease 50 (TSP50) binds to glucose‐6‐phosphate dehydrogenase (G6PD) and inhibits its acetylation at the K171 site in the cytoplasm of hepatocellular carcinoma (HCC) cells, by which TSP50 enhances lipid synthesis and promotes cell proliferation. Our study reveals a link between an oncogene and a key enzyme in HCC metabolism, which may lead to providing insight into the molecular mechanism of the PPP metabolism regulation during HCC carcinogenesis and open a new avenue for HCC treatment.

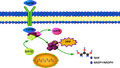

## 1 INTRODUCTION

Hepatocellular carcinoma (HCC) is one of the most common and lethal malignant tumours worldwide. Various aetiological factors including abnormal gene expression, aflatoxin absorption, excessive alcohol consumption or liver cirrhosis could cause hepatic carcinoma development and deterioration.^1^, ^2^, ^3^ One of the hallmarks of cancer cells is metabolic reprogramming and as many other tumours, HCC is known to undergo metabolic alterations to sustain faster proliferation.^4^, ^5^, ^6^, ^7^

Metabolic alterations constitute a selective advantage for tumour growth, proliferation and survival as they provide support to the crucial needs of cancer cells, such as increased energy production, macromolecular biosynthesis and maintenance in redox balance.^8^ Rapid proliferation of tumour cells requires the synthesis of raw materials and a reduction of oxidative stress.^9^ Therefore, the pentose phosphate pathway (PPP), a branch of glycolysis, is an important metabolic pathway for the survival and biosynthesis of cancer cells.^10^, ^11^ Transketolase (TKT), a key enzyme in the non‐oxidative phase of PPP, can promote the development of HCC in a non‐metabolic manner via its nuclear localization and involvement with the EGFR pathway.^12^, ^13^ A recent study demonstrates that ribose‐5‐phosphate isomerase (RPIA), a key regulator of the PPP, regulates hepatoma cell proliferation and colony formation.^14^ Glucose‐6‐phosphate dehydrogenase (G6PD), the rate‐limiting enzyme of the PPP, is elevated in many cancers and contributes to tumour growth by producing ribose‐5‐phosphate and NADPH through PPP.^15^ Elevated expression and activity of G6PD have been observed in many cancers including leukaemia, gastric cancer, renal cell carcinomas and HCC.^16^, ^17^, ^18^, ^19^ Interest in targeting G6PD as a therapeutic target in several human malignancies has increased over the last several years.^20^, ^21^ By binding to G6PD, PTEN antagonizes Tcl1/hnRNPK‐mediated G6PD pre‐mRNA splicing, thereby inhibiting hepatocarcinogenesis.^16^ Drug resistance is one of the major concerns in the treatment of HCC, and it has a significant association with the PPP in HCC.^22^

TSP50, an independent risk factor for poor prognosis in multiple cancers, promotes cancer cell proliferation and increases the expression of the epithelial‐to‐mesenchymal transition (EMT) markers.^23^, ^24^, ^25^ In addition, studies have shown that TSP50 plays a role in the invasion and metastasis of other cancer cells, including non‐small‐cell lung cancer, gastric cancer and breast cancer cells.^23^, ^26^, ^27^ A mechanistic analysis suggests that the nuclear factor κB (NF‐κB) signalling pathway is mainly involved in TSP50‐mediated cell proliferation and invasion.^28^, ^29^ Co‐immunoprecipitation studies demonstrate an interaction of TSP50 with the NF‐κB‐IκBα complex, but not with the IKK (IκB kinase) α/β‐IKKγ complex, which suggests that TSP50, as a novel protease, promotes the degradation of IκBα proteins by binding to the NF‐κB‐IκBα complex.^24^ Although several cancer‐driving mechanisms have been identified, the role of oncogenes in shaping metabolic patterns in cancer cells is only beginning to be appreciated. Recent studies have shown that oncogenes directly regulate critical metabolic enzymes and metabolic signalling pathways.^30^ However, whether TSP50 is involved in the occurrence of liver cancer by regulating metabolic abnormalities has not been well understood.

In the present research, we found that TSP50 directly binds to G6PD, inhibits the acetylation of G6PD in K171 site and promotes the formation of its active dimer, thereby increasing NADPH levels and lipid synthesis. Importantly, we further demonstrate that TSP50‐mediated inhibition of G6PD K171 acetylation is critical for its role in promoting cell proliferation, elucidating a previously unappreciated mechanism by which TSP50 contributes to HCC deterioration.

## 2 MATERIALS AND METHODS

### 2.1 Antibodies and reagents

Antibodies and reagents used in the study are as follows: anti‐TSP50 antibody was prepared and purified by Abcam (1:1000). Anti‐G6PD (25413‐1‐AP, 1:1000), anti‐GST (10000‐0‐AP, 1:1000), anti‐Flag (20543‐1‐AP, 1:1000), anti‐SIRT2 (19655‐1‐AP, 1:1000), anti‐pan acetylation antibody (66289‐1‐Ig, 1:1000), anti‐O‐glycosylation antibody (20415‐1‐AP, 1:1000), rabbit IgG, anti‐actin antibody (60008‐1‐Ig, 1:5000) and mouse IgG were purchased from Protein‐tech Group. Anti‐G6PD K171ac was designed and prepared by Absin Bioscience Inc (1:500), and anti‐KAT9 was purchased from Bioss Antibodies (bs‐20539R, 1:500). NADP+ was purchased from MedChemExpress. NADP+/NADPH detection kit and G6PD activity detection kit were purchased from Beyotime. TG and T‐CHO detection kit were purchased from Nanjing Jiancheng Bioengineering Institute. A glutaraldehyde solution was purchased from Sigma‐Aldrich.

### 2.2 Plasmid construction

To construct the gene expression plasmid, the *TSP50* and *G6PD* sequences were cloned into pEGFP‐C1 and pcDNA3.1 plasmid, respectively, for cell transfection. For knockdown, short‐hairpin RNA (shRNA) coding sequences were cloned into a pSGU6/GFP/Neo vector. The shRNA sequences knocking down TSP50 were as follows: shRNA1: GGAACTCAAGTACA GCAATTATTCAAGAGATAATTGCTGTACTTGAGTTCCTT, shRNA2: GTTCTGCTA TGAGCTAACTGGTTCAAGAGACCAGTTAGCTCATAGCAGAACTT and shRNA3: GTTCTGCTATGAGCTAACTGGTTCAAGAGACCAGTTAGCTCATAGCAGAACTT. WT G6PD and its mutants were cloned into pcDNA3.1‐Flag plasmid. pGEX‐4T1 was used for GST‐tagged protein expression in bacteria. pLVX‐AcGFP‐N1, pLVX‐AcGFP‐TSP50, pLVX‐AcGFP‐G6PD and pLVX‐AcGFP‐G6PD K171Q were constructed for lentivirus production.

### 2.3 Cell culture and plasmid transfection

L02, Huh7 and Bel7402 cells were obtained from the cell library of the Chinese Academy of Sciences. L02 cell was maintained in Roswell Park Memorial Institute (RPMI) 1640 medium supplemented with 20% foetal bovine serum (FBS). Huh7 cell and Bel7402 cell were cultured in RPMI 1640 medium supplemented with 10% foetal bovine serum (FBS). All cell lines were tested negative for mycoplasma. Twenty‐four hours before transfection, the cells were plated at a concentration of approximately 1 × 10^6^ cells/well into six‐well culture plates in a 37°C incubator with a humidified atmosphere of 5% CO_2_.

When the cell confluence reached 80%, 200 µL of RPMI 1640 medium was mixed with 5 µL of X‐tremeGENE HP (Roche) and 2 µg of plasmid, and the transfection mixture was added to each well containing the cells in medium after incubation at room temperature for 30 minutes.

### 2.4 Co‐IP‐MS/MS analysis

To screen for the proteins binding to TSP50, eluted Co‐IP samples were collected according to the Pierce cross‐link immunoprecipitation kit (Thermo: 26147) using anti‐TSP50 antibody with Huh7 cell and then the protein samples were determined by LC‐MS/MS. Candidate proteins that bind to TSP50 with specificity were obtained after non‐specific proteins were removed.

### 2.5 Western blot analysis

Protein concentration was determined using the BCA protein assay kit (Boster). Total protein (35 µg per sample) was resolved by SDS‐PAGE and transferred onto PVDF membranes (Bio‐Rad Laboratories Inc). Immunoblotting was conducted using the primary antibodies at dilutions recommended by the manufacturer. β‐actin was used as a loading control. The immunoblots were detected using an ECL advanced Western blotting detection kit (Invitrogen).

### 2.6 MTT assay

Cell viability was determined by MTT assay. A total of 2000 cells per well were seeded in a 96‐well tissue culture plate. Then, the cells of each group were transfected. 24, 36, 48 and 72 hours after transfection, MTT assays were performed using a detection kit (Beijing Solarbio Science & Technology Co., Ltd.) according to the manufacturer's protocol.

### 2.7 BrdU incorporation assay

Cell proliferation assays were performed by seeding 2000 cells per well in a 96‐well tissue culture plate. Twenty‐four hours after transfection, BrdU incorporation assay was determined by using BrdU detection kit (Solarbio). After the cells were incubated for 6 hours, labelling was stopped and BrdU uptake was measured according to the protocol of the manufacturer.

### 2.8 Triglyceride (TG) and cholesterol (T‐CHO) detection

Forty‐eight hours after transfection, the medium was removed and the cells were collected. Then, the cells were washed several times with PBS. After washing, 100 μL 0.1 mol/L PBS was used for cell sonication. Then, the TG and T‐CHO contents were measured by TG and T‐CHO detection kit (Nanjing Jiancheng Bioengineering Institute).

### 2.9 QPCR

Primers used for QPCR were designed using the Primer 5.0 gene primer design software. All primers were synthesized by Sangon Biotech Co., Ltd. and listed in Tables 1 and 2. The mRNA expression levels were detected in triplicate using the SYBR Green Ⅰ dye method. β‐actin was used as a reference gene. The RT‐PCR mixture consisted of cDNA (1 μL), PCR‐Master Mix (5 μL), PCR‐F‐Primer (0.5 μL), PCR‐R‐Primer (0.5 μL) and RNase‐free H_2_O (3 µL) in a total volume of 10 μL. The RT‐PCR conditions were as follows: 95°C denaturation for 1 minute, followed by 40 cycles of denaturation at 95°C for 15 seconds, annealing at 60°C for 15 seconds and extension at 72°C for 15 seconds. At the end of the reaction, the melting curve was recorded for the 60‐95°C temperature range, and the reaction products were stored at 4°C. The data obtained from the Eppendorf real‐time PCR instrument were analysed using the 2^−ΔΔCT^ method.

**TABLE 1:** Primer sequences of real‐time PCR

Symbol	Primer	Primer sequence (5′‐3′)
*TSP50*	F‐primer	ACAGGGAGGAGTTCTGCTATGAGATAAC
	R‐primer	AAAGATGGGTGGGGCCTCGCTCTTCTTG
*β‐actin*	F‐primer	CGTGCGTGACATTAAGGAGAAG
	R‐primer	GGAAGGAAGGCTGGAAGAGTG
*G6PD*	F‐primer	GCCAACCGCCTCTTCTAC
	R‐primer	GCGACCCTCAGTGCCAAA
*ACC*	F‐primer	CAGTGGAGCAAGAATCGG
	R‐primer	CGAGGACTTTGTTGAGGG
*FAS*	F‐primer	CTTGGTCTTCTTTATTGGCAT
	R‐primer	AGGAAAATTACAAATGGCCTT
*Fatp2*	F‐primer	CCGGTTTCTAAGAATACAGG
	R‐primer	ATCCAAGAAATACAAGGCAT
*Fatp5*	F‐primer	CCCATTTCATCCGCATCCAG
	R‐primer	TGGTACATTTCTGCCGTCA
*CD36*	F‐primer	AACCTATTGGTCAAGCCAT
	R‐primer	ATGTTTGCCTTCTCATCACC

Abbreviations: ACC, acetyl‐CoA carboxylase alpha; CD36, CD36 molecule; FAS, Fas cell surface death receptor; Fatp2, solute carrier family 27 member 2; Fatp5, solute carrier family 27 member 5; G6PD, glucose‐6‐phosphate dehydrogenase; TSP50, serine protease 50; β‐actin, cardiac muscle actin.

**TABLE 2:** Primer for G6PD mutation construction

Symbol	Primer	Primer sequence (5′‐3′)
*G6PD K89Q*	F‐primer	AGTGAGCCCTTCTTCCAGGCCACCC
	R‐primer	GGGTGGCCTGGAAGAAGGGCTCACT
*G6PD K171Q*	F‐primer	GCATCATCGTGGAGCAGCCCTTCGG
	R‐primer	CCGAAGGGCTGCTCCACGATGATGC
*G6PD K386Q*	F‐primer	CATCTTCCACCAGCAGTGCCAGCGC
	R‐primer	GCGCTGGCACTGCTGGTGGAAGATG
*G6PD K403Q*	F‐primer	GTACACCCAGATGATGACCAAGAAG
	R‐primer	CTTCTTGGTCATCATCTGGGTGTAC
*G6PD K432Q*	F‐primer	ACAGATACAAGAACGTGCAGCTCCC
	R‐primer	GGGAGCTGCACGTTCTTGTATCTGT
*G6PD K497Q*	F‐primer	GGAGGCAGACGAGCTGATGCAGAGA
	R‐primer	TCTCTGCATCAGCTCGTCTGCCTCC
*G6PD K514Q*	F‐primer	CAAGTGGGTGAACCCCCACCAGCTC
	R‐primer	GAGCTGGTGGGGGTTCACCCACTTG

Abbreviations: G6PD, glucose‐6‐phosphate dehydrogenase; K, lysine; Q, glutamine.

### 2.10 Co‐immunoprecipitation (Co‐IP)

Co‐immunoprecipitation experiments were performed using a Pierce cross‐link immunoprecipitation kit from Thermo Scientific according to the standard protocol of the manufacturer (Thermo: 26147).

### 2.11 GST pull‐down assay

GST‐TSP50 protein was extracted and purified from B21 bacteria after 0.5 mmol/L isopropyl β‐d‐1‐thiogalactopyranoside treatment at 37°C using a GST‐Sefinose kit (Sangon Biotech, C600327). Meanwhile, Flag‐G6PD protein was extracted from Huh7 cell or Bel7402 cell lysates by IP. Then, GST‐TSP50 was incubated with GST beads for 3 hours. Next, the eukaryotic expression protein Flag‐G6PD was added to the mixture and the column was rotated vertically on the mixer (Grant: PTR‐35) for 3 hours. Then, 500 μL of 1× PBS was used to wash the protein mixture five times to completely remove unbound proteins. After washing, 100 μL lysis buffer was used to resuspend the pellets and the lysates were used for Western blot assay.

### 2.12 Immunofluorescence and confocal laser scanning microscopy

Cells were pre‐seeded 1 day before immunofluorescence analysis when the culture on coverslips reached a final confluence of 70%‐80%. The cells were fixed in 4% paraformaldehyde for 10 minutes, permeabilized with 0.1% Triton X‐100 for 5 minutes, blocked with 5% bovine serum albumin and incubated with the indicated antibodies, followed by a Texas‐red‐conjugated anti‐rabbit IgG and a fluorescein isothiocyanate–conjugated anti‐mouse IgG antibody. The cells were mounted with DAPI‐containing medium (Vector Laboratories), and images were acquired with a laser scanning confocal microscope (Olympus).

### 2.13 G6PD purification and enzyme activity of the G6PD assay

G6PD enzyme activity was determined by using a G6PD activity assay kit purchased from Beyotime Institute of Biotechnology according to the manufacturer's instructions. Enzyme activities were normalized on the basis of protein concentration, which was determined by the Bradford method.

### 2.14 Glutaraldehyde cross‐linking

Glutaraldehyde cross‐linking was used to detect the G6PD dimer. Briefly, cells were trypsinized and counted. Equal numbers of cells were collected for the experiment. Cells were washed with cold PBS, followed by suspension in 0.25% glutaraldehyde buffer. After incubating at 37°C for 30 minutes, the samples were boiled and used for Western blot assay.

### 2.15 Immunoprecipitation

Huh7 cell or Bel7402 cell was lysed in buffer containing 20 mmol/L HEPES (pH 7.5), 150 mmol/L NaCl, 2 mmol/L EDTA, 1.5 mmol/L MgCl_2_, 1% NP40 and protease inhibitors for 1 hour at 4°C followed by centrifugation. The supernatants were then diluted in a buffer without NP40 and pre‐cleared by protein A/G‐Sepharose beads for 30 minutes. The supernatants were then incubated with indicated antibody for 4‐6 hours at 4°C, followed by incubation with protein A/G‐Sepharose beads for 1 hour at 4°C. After incubation, the beads were washed five times with lysis buffer, followed by further washing with ice‐cold PBS and boiling in 2× loading buffer. The protein samples were resolved with SDS‐PAGE.

### 2.16 RNA isolation and RNA sequencing

TRIzol reagent was used to extract total RNA. Then, the RNA concentration was detected using a spectrometer (Malcom: e‐spect). The amount of the precipitated mRNA was normalized to the input RNA fractions to eliminate possible differences in RNA sample preparation. RNA sequencing was performed by the Beijing Genomics Institute (BGI). Gene was considered significant when the Hochberg‐adjusted *P* value (*P*_adj._) was <.05 and the differentially expressed genes (DEGs) were subjected for enrichment analysis by the Gene Ontology (GO) and KEGG (Kyoto Encyclopedia of Genes and Genomes) tools. The RNA‐seq raw data were deposited to ArrayExpress under the accession number E‐MTAB‐9712.

### 2.17 Tumour xenograft studies

All animal studies were conducted with approval from the Institutional Animal Care and Use Committee of Northeast Normal University (NENU/IACUC, AP20191225) of China. Female BALB/c nude mice (4‐6 weeks old) were purchased from Charles River Animal Company of China and were randomly assigned to experimental groups (n = 6 in each group). For xenograft experiments, L02 cell was infected with various lentiviruses expressing proteins of interest. Equal numbers of the established stable cell were injected subcutaneously into nude mice. Twenty‐eight days after injection, tumour volumes and weight were measured with a calliper and calculated using the equation, volume = 1/2 × *ab* ^2^ (*a* = length, *b* = width).

### 2.18 TCGA analysis

In the TCGA database, samples of hepatocellular carcinoma were selected for an analysis of TSP50 and G6PD expression. In parallel, the relationship of TSP50 and G6PD with pathological stages was ascertained using the Gene Expression Profiling Interactive Analysis (GEPIA) database (http://gepia.cancer‐pku.cn/). Then, GEPIA was used to perform survival analysis based on 182 samples with low expression of TSP50 or G6PD and 182 samples with high expression of TSP50 or G6PD. The Kaplan‐Meier method was used for statistical analysis.

### 2.19 Statistical analysis

The data were presented as the means ± SD. All data represent three independent experiments. Two‐sided Student's *t* test, one‐way ANOVA or Kaplan‐Meier analysis was used to calculate *P*‐values. Significance is displayed as *P < .05 or **P < .01.

## 3 RESULTS

### 3.1 TSP50 enhances hepatocyte proliferation

**FIGURE 1:**
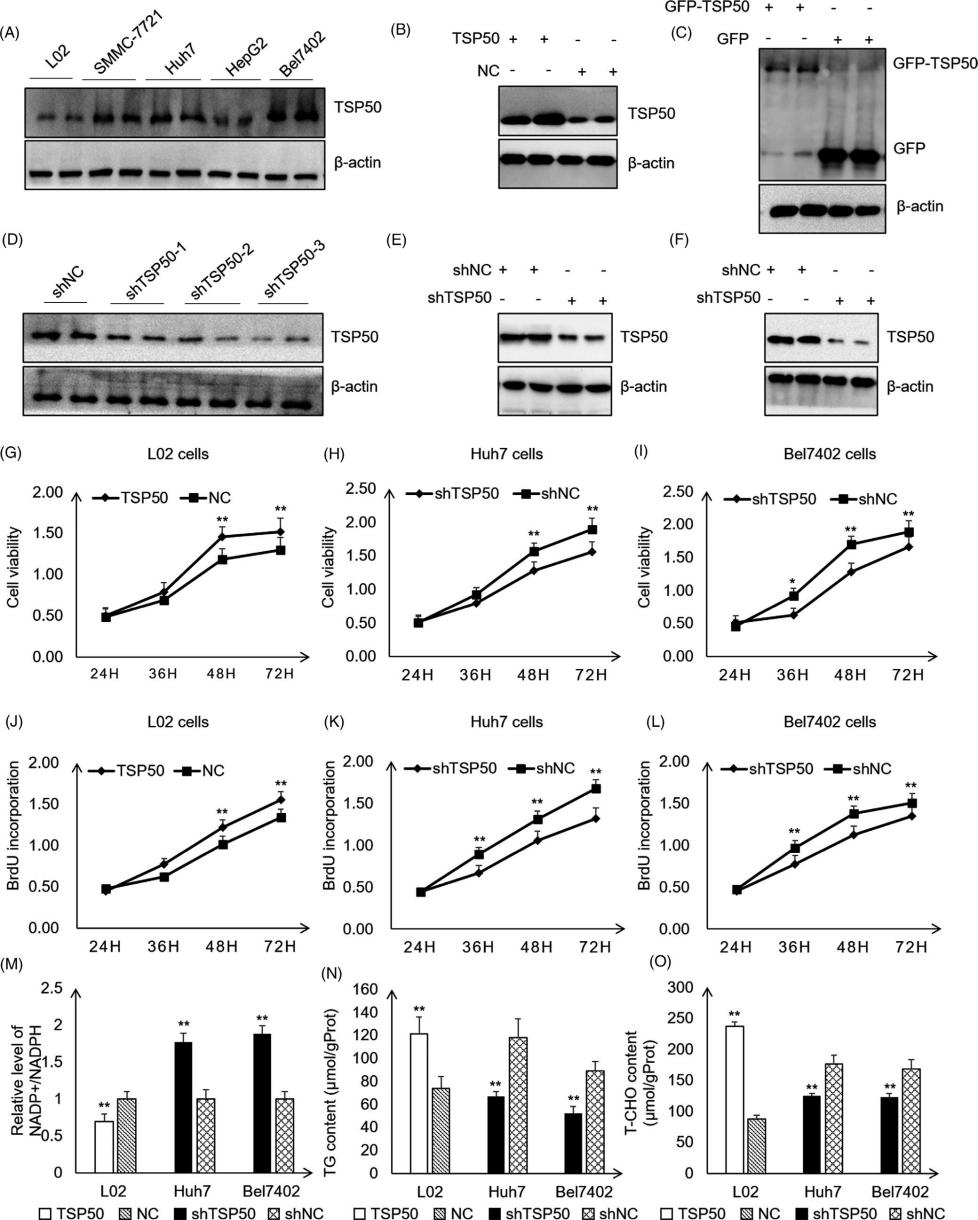
TSP50 enhances hepatocyte proliferation. A, Multiple cancer cells were cultured, harvested and subjected to Western blot analysis for TSP50 detection. B and C, PE‐GFP‐TSP50 and pcDNA3.1‐TSP50 were transfected into L02 cell to overexpress TSP50, and efficient expression of TSP50 was examined by Western blotting. D, Various of shTSP50 vectors were transfected into Huh7 cell for knockdown efficiency analysis. E and F, shTSP50 was transfected into Huh7 cell and Bel7402 cell for the gene knockdown, and the knockdown efficiency was evaluated. β‐actin served as loading control. G‐I, The cell viability was detected after pcDNA3.1‐TSP50 or shTSP50. J‐L, BrdU incorporation assay was detected in cells after pcDNA3.1‐TSP50 or shTSP50 transfection. M, NADP+/NADPH ratios were measured in L02, Huh7 and Bel7402 cells using a NADP+/NADPH assay kit (Beyotime Institute of Biotechnology. N‐O, Cells with the overexpression or knockdown of TSP50 were harvested and analysed for the intracellular levels of TG and T‐CHO. n = 3 biologically independent replicates. Data were presented as means ± SD. *P < .05 as compared to NC group by two‐sided Student's *t* test. **P < .01

To explore the functional role of TSP50 in hepatic carcinoma cells, we first detected the expression of TSP50 in human embryonic hepatocytes (L02 cell) and various HCC cell lines (SMMC‐7721 cell, Huh7 cell, HepG2 cell and Bel7402 cell). Our results showed that TSP50 is highly expressed in hepatic carcinoma cells, especially in Huh7 cell and Bel7402 cell, while the level of TSP50 is low in L02 cell, indicating that TSP50 may play a key role in HCC progression (Figure 1A) (Figure S1A). To analyse the role of TSP50 in hepatic carcinoma cells, pcDNA3.1‐TSP50 and PE‐GFP‐TSP50 were transfected into L02 cell for TSP50 overexpression, pSGU6/GFP/Neo‐shTSP50 were transfected into Huh7 cell and Bel7402 cell for TSP50 knockdown, and efficient TSP50 overexpression and knockdown were confirmed by Western blotting (Figure 1B‐F) (Figure S1B‐F). The results from the MTT assay verified that the overexpression of TSP50 promotes L02 cell proliferation, while the knockdown of TSP50 significantly inhibits the proliferation of Huh7 and Bel7402 cells (Figure 1G‐I). In addition, BrdU uptake is significantly elevated in the L02 cell overexpressing TSP50 (Figure 1J), while BrdU uptake is significantly reduced in the Huh7 and Bel7402 cells after the downregulation of TSP50 (Figure 1K,L). The results indicated that TSP50 is important for the proliferation of hepatocytes. Previous studies have shown that glucose metabolism reprogramming plays a critical role in biosynthesis in cancer cells^31^ and altered lipid metabolism is among the most prominent metabolic alterations in cancer. The enhanced synthesis or uptake of lipids contributes to rapid cancer cell growth and tumour formation.^32^ To investigate whether the proliferation‐promoting effect of TSP50 is accompanied by changes in metabolic reprogramming, we analysed the effects of TSP50 on lipid synthesis. We observed that overexpression of TSP50 significantly reduces the proportion of NADP+/NADPH and increased cholesterol (T‐CHO) and triglyceride (TG) production. In contrast, shTSP50 increases the proportion of NADP+/NADPH and inhibits T‐CHO and TG production (Figure 1M‐O). Therefore, we speculate that the increased cell proliferation induced by TSP50 may be closely correlated with the production of lipids and NADPH.

### 3.2 TSP50 binds directly to G6PD and co‐localizes with G6PD in the cytoplasm

**FIGURE 2:**
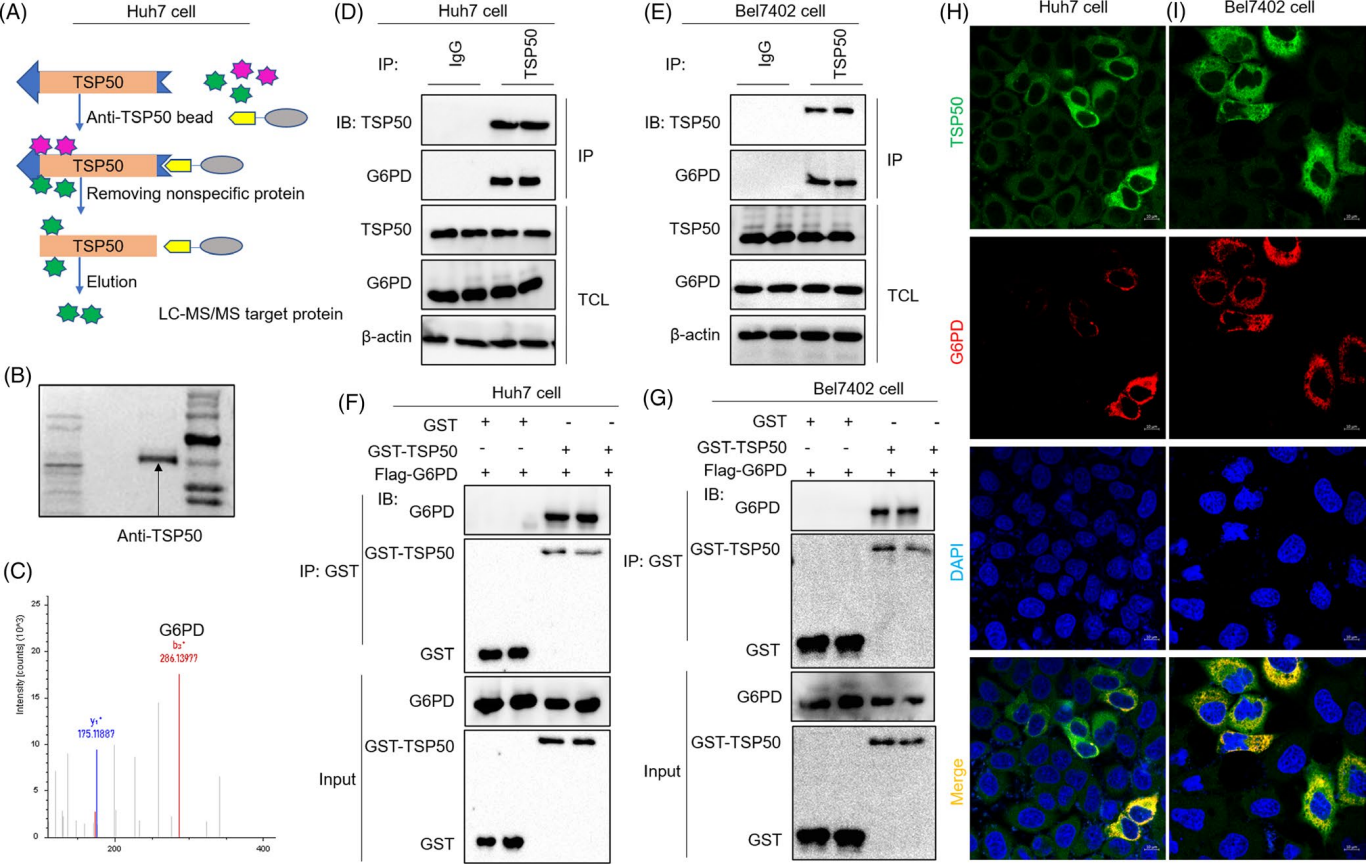
TSP50 binds directly to G6PD and co‐localizes with G6PD in the cytoplasm. A‐C, Huh7 cell was cultured and harvested, and after cell incubation with an anti‐TSP50 antibody, proteins binding to endogenous TSP50 were analysed by LC‐MS/MS. Candidate binding proteins to TSP50 were obtained after non‐specific proteins were removed. D and E, Huh7 cell and Bel7402 cell were harvested and subjected to immunoprecipitation with anti‐TSP50 antibody, followed by Western blot analysis with anti‐G6PD antibody. F and G, GST pull‐down of Flag‐G6PD by GST‐TSP50 using proteins purified in B21 bacteria, followed by Western blot analysis with anti‐G6PD and anti‐GST antibodies. H and I, Huh7 and Bel7402 cells were fixed and subjected to immunofluorescence analysis with anti‐TSP50 antibody or anti‐G6PD antibody for G6PD and TSP50 localization analysis

Next, we sought to determine the molecular mechanisms by which TSP50 promotes cell proliferation and alters lipid metabolism. Tandem mass spectrometry (MS/MS)–based proteomics combined with co‐immunoprecipitation (Co‐IP) has emerged as a powerful approach for studying protein complexes^33^; therefore, we used Co‐IP‐MS/MS for screening proteins binding to TSP50. We found that multiple proteins can interact with TSP50, of which G6PD is the only one related to NAPDH production and lipid synthesis (Figure 2A‐C). Glucose‐6‐phosphate dehydrogenase (G6PD), the rate‐limiting enzyme of the PPP, is previously reported to be elevated in HCC and contributes to tumour growth by producing ribose‐5‐phosphate and NADPH through the PPP.^15^ Next, we further examined the endogenous interaction of TSP50 with G6PD in the Huh7 cell and Bel7402 cell. As expected, co‐immunoprecipitation experiments showed that there is a specific interaction between TSP50 and G6PD in Huh7 and Bel7402 cells (Figure 2D,E). In addition, a GST pull‐down assay using purified recombinant proteins demonstrates the direct interaction between GST‐TSP50 and Flag‐G6PD (Figure 2F,G). Moreover, the immunofluorescence results further confirmed that these two proteins are co‐localized in the cytoplasm in Huh7 and Bel7402 cells (Figure 2H,I). The cytoplasm is a key location for the pentose phosphate pathway and tends to preferentially export more sugar metabolism‐associated proteins via exosomes.^34^ We speculated that TSP50 regulates PPP pathway mainly by interacting with G6PD.

### 3.3 TSP50 inhibits G6PD acetylation and increases G6PD activity

**FIGURE 3:**
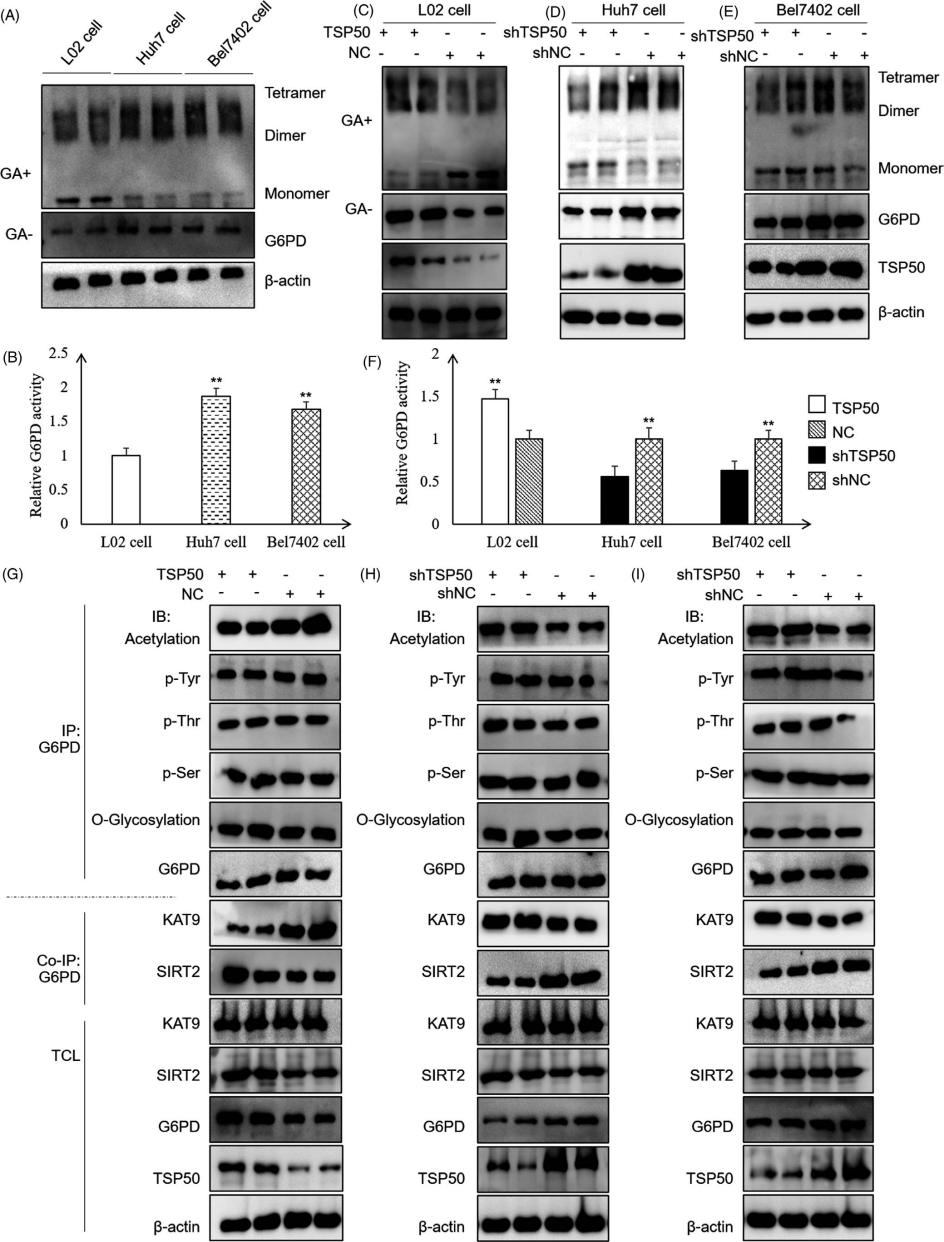
TSP50 inhibits G6PD acetylation and increases G6PD activity. A and B, For G6PD function analysis, cross‐linking and G6PD activity kits were used for endogenous G6PD expression and enzyme activity analysis of L02, Huh7 and Bel7402 cells. C‐F, The expressions and activity of G6PD were detected in the L02 cell with TSP50 overexpression and the Huh7 and Bel7402 cells with TSP50 knockdown. L02 cell and Huh7 cell or Bel7402 cell were transfected with pcDNA3.1‐TSP50 and shTSP50, respectively, before the cells were treated with or without glutaraldehyde. Subsequently, the proteins were harvested for Western blot analysis and the relative G6PD activity was detected using G6PD detection kit. G‐I, L02, Huh7 and Bel7402 cells were harvested after transfection, followed by IP with anti‐G6PD antibody and Western blot analysis with antibodies against acetylated G6PD, phosphorylated G6PD (p‐Tyr, p‐Thr and p‐Ser) and O‐glycosylation. Data were presented as means ± SD. *P <.05 as compared to NC group by one‐way ANOVA. **P < .01

It was reported that both dimers and tetramers are active forms of G6PD.^35^ To identify the expression and activity of G6PD in L02, Huh7 and Bel7402 cells, Western blot and glutaraldehyde cross‐linking assays were performed. Compared with that in the L02 cell, G6PD is highly expressed in the Huh7 and Bel7402 cells. In addition, the cross‐linking assay results showed that dimers and tetramers are the main forms of G6PD in the Huh7 and Bel7402 cells (Figure 3A) (Figure S2A‐C). Consistent with its elevated expression and the relative abundance of higher order multimers, G6PD activity is higher in the hepatoma cell lines than in L02 cell (Figure 3B).

To further explore the impact of TSP50 on G6PD, we transfected pcDNA3.1‐TSP50 into L02 cell and found that the overexpression of TSP50 leads to increased G6PD expression and enzyme activity. In contrast, the expression of G6PD is significantly reduced in the Huh7 cell and Bel7402 cell after the knockdown of TSP50 by shTSP50 (Figure 3C‐E). The data from cross‐linking experiments further verified that TSP50 promotes the formation of G6PD dimers and tetramers and greatly increases the activity of G6PD (Figure 3C‐F) (Figure S2D‐F). To further elucidate the mechanism by which TSP50 affected G6PD activity, samples were collected after immunoprecipitation and the effect of TSP50 on G6PD protein modification including acetylation, phosphorylation and O‐glycosylation was examined. Our results suggested that the overexpression of TSP50 can inhibit the acetylation of G6PD but have little effect on p‐Tyr, p‐Thr, p‐Ser and O‐glycosylation of G6PD. These results indicated that TSP50 may regulate the activity of G6PD by inhibiting its acetylation. Moreover, co‐immunoprecipitation assay demonstrated that TSP50 can inhibit the binding of the G6PD protein to acetylase KAT9 while promoting its binding to deacetylase SIRT2 in hepatoma cells (Figure 3G‐I) (Figure S2G‐I).

### 3.4 K171 is a key site for G6PD acetylation regulated by TSP50

**FIGURE 4:**
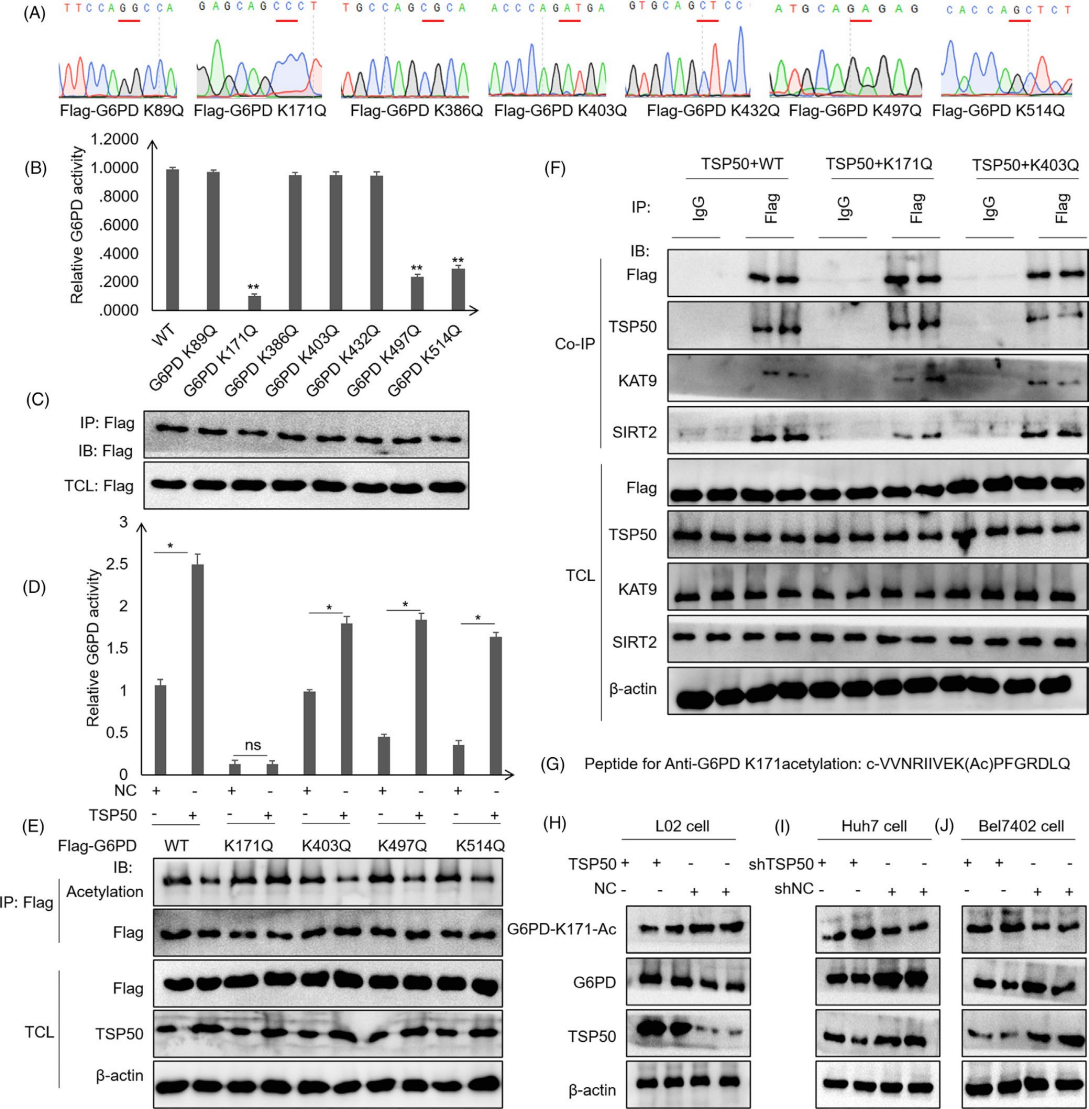
K171 is a key site for TSP50 regulation of G6PD acetylation. A, Vectors encoding for multiple mutant G6PD were constructed using a site‐directed mutagenesis kit. B‐E, The effects of TSP50 on G6PD acetylation and enzyme activity were analysed by Western blot analysis and G6PD assay after pcDNA3.1‐TSP50 or shTSP50 transfection. F, Co‐immunoprecipitation was performed to analyse the effect of K171 and K403 site mutations on the binding of G6PD to KAT9 and SIRT2. G‐J, The level of G6PD K171 acetylation was detected using an anti‐G6PD K171ac antibody

To explore the G6PD acetylation site targeted by TSP50, multiple G6PD mutants including K89Q, K171Q, K386Q, K403Q, K432Q, K497Q and K514Q were constructed and conserved acetylated sites in G6PD regulated by TSP50 were screened (Figure 4A). The G6PD protein was purified by immunoprecipitation using anti‐Flag antibody, and the activity of G6PD was tested. Compared with the effects after wild‐type vector transfection, G6PD activity is significantly decreased in cell after K171Q, K497Q and K514Q vector transfection, suggesting that the acetylation of these sites is a critical factor affecting the enzyme activity of G6PD (Figure 4B,C) (Figure S3A). More importantly, the overexpression of TSP50 does not alter G6PD enzyme activity in cell that had been transfected with G6PD K171Q (Figure 4D,E) (Figure S3B‐E), indicating that TSP50 enhances G6PD activity mainly by regulating G6PD K171 acetylation. To further analyse the mechanism by which TSP50 regulated G6PD acetylation, a co‐immunoprecipitation assay was performed, and the results showed that TSP50 promotes the binding of G6PD to SIRT2 but not to KAT9 (Figure 4F). To further support these results, we designed peptides for the acetylation of G6PD K171 and immunized rabbits with them to obtain antibody against G6PD with K171 acetylation (Figure 4G). Remarkably, pcDNA3.1‐TSP50 transfection inhibits the acetylation of G6PD K171 in L02 cell, while shTSP50 up‐regulates the acetylation of G6PD K171 in Huh7 and Bel702 cells (Figure 4H‐J) (Figure S3F‐H). Collectively, these results suggested that TSP50‐mediated deacetylation of G6PD at K171 is an important tumorigenic factor in liver cancer.

### 3.5 TSP50 promotes hepatocyte proliferation and lipid metabolism by accelerating G6PD activity

**FIGURE 5:**
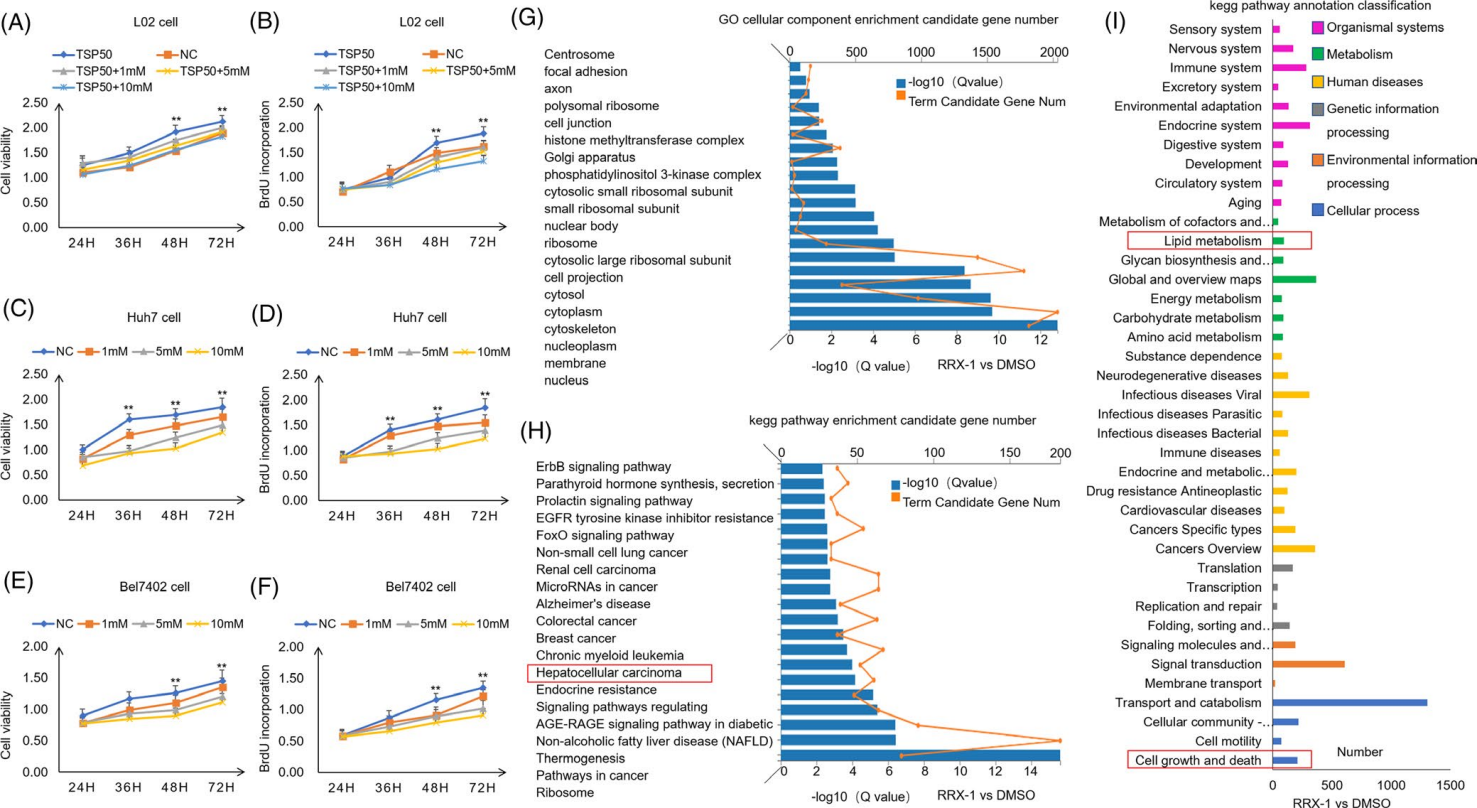
The change in G6PD enzyme activity modifies metabolic reprogramming in liver cancer cells. A‐F, Cell viability and cell proliferation were detected in the presence or absence of NADP+ by MTT assay and BrdU incorporation assay. G‐I, After cell was treated with 20 μmol/L G6PD enzyme activity inhibitor RRx‐001 for 24 hours, RNA was harvested for RNA sequencing. The genes of interest were selected based on significantly differential expression in the RNA‐seq analysis. GO and KEGG analyses were conducted using the DAVID bioinformatics database

Since G6PD directly reduced the ratio of NADP+/NADPH which may be directly involved in the proliferation of liver cancer cells, we evaluated the effect of NADP+ on cell proliferation. The addition of NADP+ reverses the proliferative effect of TSP50 in L02 cell (Figure 5A,B). Moreover, the results showed that NADP+ inhibits Huh7 and Bel7402 cell proliferation in a concentration‐dependent manner (Figure 5C‐F). In order to further determine that the increased G6PD enzyme activity mediated by TSP50 is involved in metabolic reprogramming in liver cancer cells, RNA sequencing was performed and the results showed that the addition of the G6PD inhibitor RRx‐1 alters the expression levels of a large number of genes. Gene ontology analysis showed that inhibition of G6PD activity led to changes in lipid‐related genes, including *ACC*, *GPD2*, *LSS*, *KANSL1L*, *LPCAT1*, *ZSCAN25*, *SULT2B1*, *PNPLA7*, *ACSL1*, *CPT1C*, *FATP2*, *ZNF37A*, *HACD2*, *ACSL4*, *GPAM*, *ACACA*, *FASN*, *UGT8*, *SCD* and *FAR1* (Figure 5G). In addition, KEGG pathway analysis showed that RRx‐1 addition can lead to liver carcinogenesis and nonalcoholic fatty liver disease and enrichment with signalling pathways related to metabolic reprogramming in Huh7 cell (Figure 5H,I). These results further validate the important role of G6PD enzyme activity in liver carcinogenesis and metabolic reprogramming.

**FIGURE 6:**
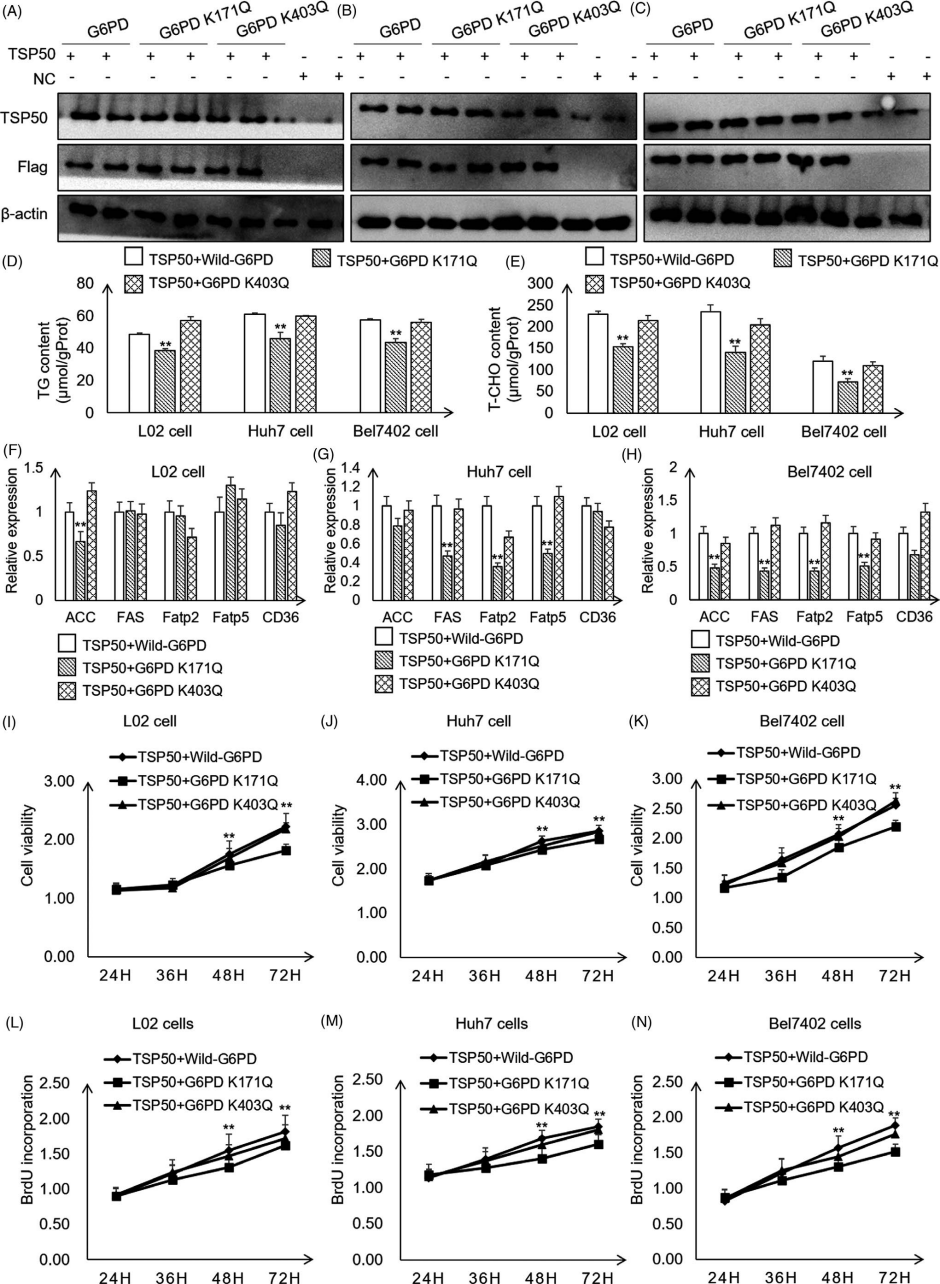
TSP50 promotes hepatocyte proliferation and lipid metabolism by mediating G6PD activity. A‐C, pcDNA3.1‐TSP50 in combination with Flag‐G6PD, Flag‐G6PD K171Q or Flag‐G6PD K403Q was co‐transfected into L02, Huh7 and Bel7402 cells to overexpress TSP50 and G6PD wild‐type or mutants’ vectors. The efficient expression of TSP50 or G6PD (wild type and mutants) was examined by Western blot analysis using anti‐TSP50 and anti‐Flag antibodies, respectively. D‐E, TG and T‐CHO contents were detected in cells that co‐transfected with TSP50 and G6PD or G6PD K171Q or G6PD K403Q. F‐H, Relative expression of lipid metabolism–related genes was detected by RT‐PCR assay. I‐K, Cell viability was detected by MTT assay after co‐transfection of pcDNA3.1‐TSP50 with Flag‐G6PD or Flag‐G6PD mutants in L02, Huh7 and Bel7402 cells. L‐N, The BrdU incorporation assay was performed in cells after co‐transfection of pcDNA3.1‐TSP50 with Flag‐G6PD or Flag‐G6PD mutants. Data were presented as mean ± SD. *P < .05 as compared to NC group by one‐way ANOVA. **P < .01

Next, we examined whether acetylation of G6PD at the K171 site mediated by TSP50 is related to cell proliferation and metabolic changes, and pcDNA3.1‐TSP50 was co‐transfected with pcDNA‐3.1‐Flag‐G6PD, pcDNA‐3.1‐Flag‐G6PD K171Q and pcDNA‐3.1‐Flag‐G6PD K403Q into L02, Huh7 and Bel7402 cells (Figure 6A‐C) (Figure S4A‐F). We found that the co‐transfection of pcDNA3.1‐TSP50 with pcDNA‐3.1‐Flag‐G6PD K171Q leads to lower TG content and T‐CHO production (Figure 6D,E). Furthermore, co‐transfection of pcDNA3.1‐TSP50 with Flag‐G6PD K171Q significantly inhibits the expression of *ACC*, *FAS*, *Fatp2* and *CD36* in these cells (Figure 6F‐H). Interestingly, the MTT assay revealed that the co‐transfection of pcDNA3.1‐TSP50 with Flag‐G6PD K171Q reduces cell proliferation than compared with its effect in the in L02, Huh7 and Bel7402 cells transfected with wild‐type or K403Q constructs (Figure 6I‐K). Furthermore, we found that co‐transfection of pcDNA3.1‐TSP50 with pcDNA‐3.1‐Flag‐G6PD K171Q leads to lower BrdU uptake (Figure 6L‐N). These data suggested that G6PD‐mediated metabolic changes are important for TSP50‐induced cell proliferation. Taken together, these results suggest that the effect of TSP50 on hepatocyte proliferation depends at least partially on G6PD activity.

### 3.6 TSP50 promotes tumour formation by inhibiting G6PD K171 acetylation

**FIGURE 7:**
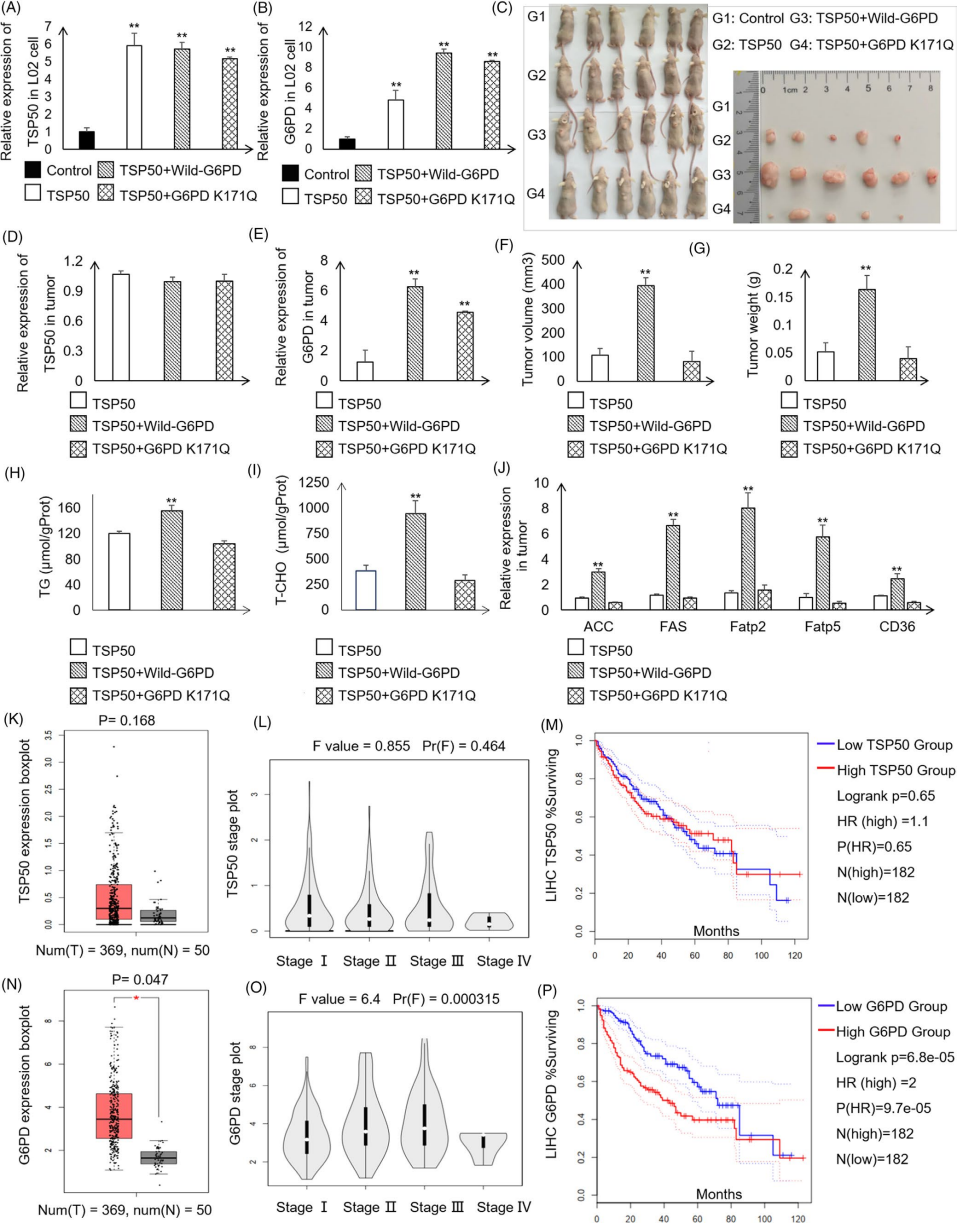
TSP50 and G6PD promote tumour formation and correlate with HCC patient survival. A‐C, Photography of xenograft tumour‐bearing mice injected with different cell lines. D and E, Expression of TSP50 and G6PD in tumours determined by RT‐PCR. F and G, Tumour volume and tumour weight for each group. H and I, T‐CHO and TG contents in tumour tissue were detected in each group. J, Expression of genes related to lipid metabolism determined by RT‐PCR. K, TSP50 expression in HCC tissue and normal control tissue based on the TCGA database. L, Violin plots for the correlation of TSP50 with HCC stages based on the Gene Expression Profiling Interactive Analysis (GEPIA) database (http://gepia.cancer‐pku.cn/). M, The correlation between TSP50 expression and HCC patient survival in the TCGA cohort. Patients were classified into TSP50‐low and TSP50‐high expression groups based on median mRNA expression (n = 364). N, G6PD expression in HCC tissue and normal control tissue based on the TCGA database. O, Violin plots for the correlation of G6PD with HCC stages. P, The correlation between G6PD expression and HCC patient survival in the TCGA cohort. The patients were classified into G6PD‐low and G6PD‐high expression groups based on median mRNA expression (n = 364). *P < .05, **P < .01

To address whether TSP50‐mediated G6PD activity changes are important for tumour growth, we performed xenograft experiments using L02 cell. We constructed cells stably expressing TSP50, G6PD and G6PD K171Q by lentivirus infection, and cell expressing these proteins was injected subcutaneously into nude mice (Figure 7A,B).

After 4‐week in vivo tumour growth, the mice were sacrificed, and tumour growth was assessed. Compared with the pLVX‐AcGFP‐N1 group, the overexpression of TSP50 greatly promotes tumour formation (Figure 7C). The relative expression of TSP50 and G6PD is consistent with that in tumour cells (Figure 7D,E). More importantly, tumour growth is remarkably inhibited after the expression of G6PD K171Q in the TSP50‐expressing cell compared to that in G6PD wild‐type group. Additionally, the tumour size and tumour weight are decreased in the mice injected with cell expressing G6PD K171Q (Figure 7F,G). The result showed that overexpression of TSP50 obviously enhances growth of G6PD wild‐type cell; however, it has no effect on the G6PD K171 mutated cell. The T‐CHO and TG contents were measured in tumour tissue, and the results showed that the levels of T‐CHO and TG in TSP50 and wild‐type G6PD‐expressing group are significantly higher than those in other two groups (Figure 7H,I). In addition, the relative expression levels of *ACC*, *FAS*, *Fatp2*, *Fatp5* and *CD36* are higher in the TSP50 and wild‐type G6PD‐expressing groups (Figure 7J). These results underline the importance of the acetylation of G6PD as regulated by TSP50 in tumour development. Taken together, these results demonstrate that G6PD and its activity regulated by acetylation and deacetylation are critical for TSP50‐mediated tumour growth in vitro and in vivo.

### 3.7 The expression of TSP50 and G6PD is closely associated with the survival of hepatocellular carcinoma patients

Considering these results, we concluded that TSP50 and G6PD expression is associated with hepatocyte proliferation and xenograft tumour formation. To validate their possible effects on patient survival, we analysed the data from The Cancer Genome Atlas (TCGA). In HCC‐control tissue pairs (n = 50), both TSP50 and G6PD are highly expressed in HCC tissue (n = 369) (Figure 7K,N). Therefore, we focused on the expression of TSP50 and G6PD in patients with HCC. Interestingly, the expression of TSP50 and G6PD differs in various stages (Figure 7L,O). Next, 182 samples with low expression and 182 samples with high expression of TSP50 and G6PD were selected for survival analysis. Remarkably, high expression of TSP50 (HR = 1.1) and G6PD (HR = 2) is closely correlated with poor survival of HCC patients, suggesting their potential link with the progression of the disease (Figure 7M,P). The results further illustrate the importance of abnormal G6PD and TSP50 expression in HCC progression.

## 4 DISCUSSION

Increasing evidence points to pivotal roles for metabolic reprogramming in regulating a diverse set of processes that function in cell proliferation and cell cycle changes, especially in cancer cells.^36^, ^37^, ^38^ In the current paradigm, aerobic glycolysis is considered the central metabolic characteristic of cancer cells (Warburg effect). However, recent data indicate that cancer cells also show significant changes in other metabolic pathways. Indeed, it is recently suggested that the Krebs cycle, pentose phosphate pathway intermediates, and essential and nonessential amino acids play key roles.^39^ As a rate‐limiting enzyme in PPP, G6PD participates in glucose utilization by catalysing the first step of the PPP in a variety of cancer cells, including the A375 melanoma cell line and the Hep3B hepatocellular carcinoma line.^22^, ^40^ Activation of G6PD by SIRT2 supports the proliferation and clone formation of leukaemia cell.^41^ G6PD may be regulated apoptosis and expression of cell cycle–related proteins through phosphorylation of transcription factors STAT3 and STAT5, thus mediating formation and growth of human melanoma cells.^42^ In the present study, we found that G6PD is highly expressed in Huh7 and Bel7402 cells and that the highly active form of G6PD promotes hepatocyte proliferation and tumour formation. We speculate that, on the one hand, G6PD increases more materials needed for cell proliferation, and on the other hand, it effectively reduces intracellular oxidative stress by balancing NADP+ and NADPH.

Here we established that the activities of the metabolic enzyme G6PD are regulated by TSP50 in hepatoma cell lines. Our results demonstrated that TSP50 not only promotes the expression of G6PD in hepatocytes, but also regulates its activity by mediating G6PD acetylation. Consistent with the present observations, previous study demonstrated that exposure of HCT116 human colorectal cancer cell to aspirin causes the acetylation of G6PD, and this is associated with a decrease in its enzyme activity.^43^ In addition, activation of G6PD by SIRT2 supports the proliferation of leukaemia cell.^41^ G6PD was identified to be acetylated on seven lysine residues, including lysine 89 (K89), lysine 171 (K171), lysine 386 (K386), lysine 403 (K403), lysine 432 (K432), lysine 497 (K497) and lysine 514 (K514),^44^ with lysine 171 being key site that directly impacts the enzyme activity of G6PD.^45^ In contrast to other studies, we found that the effect of TSP50 on cell proliferation is mediated by the inhibition of the acetylation of the G6PD K171 site in hepatoma cell lines, which is novel finding of this study. Interestingly, Wang et al proved that the deacetylase SIRT2 promotes NADPH production by deacetylating G6PD at lysine 403 (K403) and that G6PD K403 deacetylation and activation act protection against oxidative stress in vivo.^45^ To confirm our results, we designed a peptide to prepare an anti‐G6PD K171 acetylation antibody. The results proved that TSP50 directly regulates the acetylation of the G6PD K171 site in Huh7 and Bel7402 cells. We speculate that the difference in acetylation sites may be related to the type of cell.

TSP50, a novel identified oncogene, has been reported to induce several cell proliferation and tumour formation.^46^, ^47^ Detailed mechanism analysis found that TSP50‐induced cell proliferation may be related to NF‐κB activity.^24^ The difference is that we illustrate the cancer‐promoting mechanism of TSP50 in a new perspective. In the present study, we found that TSP50 affects the acetylation of G6PD by regulating the binding of G6PD to KAT9 and SIRT2. According to the co‐immunoprecipitation analysis, knocking down TSP50 promotes the binding of G6PD to KAT9 and inhibits its binding to SIRT2. SIRT2‐mediated deacetylation and activation of G6PD stimulate the PPP to supply cytosolic NADPH to counteract oxidative damage and protect mouse erythrocytes.^45^ Therefore, we conclude that TSP50 is a key factor in promoting the binding of G6PD to deacetylation‐related enzymes to form functional complexes, thereby regulating G6PD activity. However, the limitation of this model is that currently, there is no mechanistic evidence to explain why TSP50 preferentially binds to SIRT2 instead of KAT9. The determination of the exact mechanism for this binding preference by TSP50 will require further structural and biochemical studies.

The increased TG and T‐CHO content induced by TSP50 through PPP pathway may be critical for the enhanced hepatocyte proliferation and tumour formation. Lin et al^48^ demonstrated that suppression of 6PGD decreases lipogenesis and RNA biosynthesis and elevated ROS levels in cancer cells, attenuating cell proliferation and tumour growth. Consistent with this finding, ZHX2 inhibits uptake of exogenous lipids through transcriptional suppression of lipid lipase (LPL), which result in delaying the proliferation of HCC cells. Importantly, LPL overexpression significantly reverses ZHX2‐mediated inhibition of HCC cell proliferation, xenograft tumour growth, lipid deposition and spontaneous liver tumour formation.^49^ Therefore, we believe that increased lipid synthesis provides the raw material for rapid cell proliferation. To verify that increased lipid synthesis is necessary for TSP50‐induced cell proliferation, we plan to supplement cells with various exogenous lipids and investigate whether they can rescue cell proliferation in TSP50‐knockdown cell lines in future research. Additionally, we found that the overexpression of TSP50 mainly increases *ACC* expression in L02 cell, while overexpression of TSP50 greatly increases *ACC*, *FAS*, *Fatp5* and *Fatp2* expression in Huh7 or Bel7402 cells. This difference may be due to the differences in lipid metabolism between normal and cancer cells, and future investigations will be required to identify the potential mechanism.

Glucose 6‐phosphate dehydrogenase (G6PD) is essential for the maintenance of nicotinamide dinucleotide hydrogen phosphate (NADPH) levels and redox homeostasis in physiological processes.^7^ Therefore, we believe that it may be more reasonable to study the inhibitors of TSP50 instead of G6PD for the treatment of HCC, especially for female patients. In the future, we will investigate the therapeutic effects of small‐molecule compounds on liver cancer and develop new small‐molecule compounds specifically targeting TSP50. Our finding that TSP50 positively regulates G6PD activity adds new mechanistic insight into the regulation of PPP and suggests that modulating TSP50‐mediated G6PD activity may be a potential therapeutic strategy for liver cancer.

## CONFLICT OF INTEREST

None.

## AUTHOR CONTRIBUTIONS

Xiaojun Zhang and Feng Gao designed and performed experiments, analysed the data and wrote the manuscript. Huihan Ai and Shuyue Wang designed and performed experiments and analysed the data. Lihua Zheng, Guannan Wang and Ying Sun performed bioinformatics analyses. Zhenbo Song and Yongli Bao conceived the overall project and participated in experimental design, data analyses, interpretations and manuscript writing.

## Supporting information

Supplemental information can be found deposited online in ArrayExpress with accession number E‐MTAB‐9712.

Fig S1

Fig S2

Fig S3

Fig S4

## Data Availability

The data that support the findings of this study are available from the corresponding author upon reasonable request.
