# Supplementary figures and images for: TSP50 promotes hepatocyte proliferation and tumour formation by activating glucose‐6‐phosphate dehydrogenase (G6PD)
Source: Cell Prolif. 2021 Feb 25;54(4):e13015. doi: 10.1111/cpr.13015 (PMC8016650; doi:10.1111/cpr.13015)

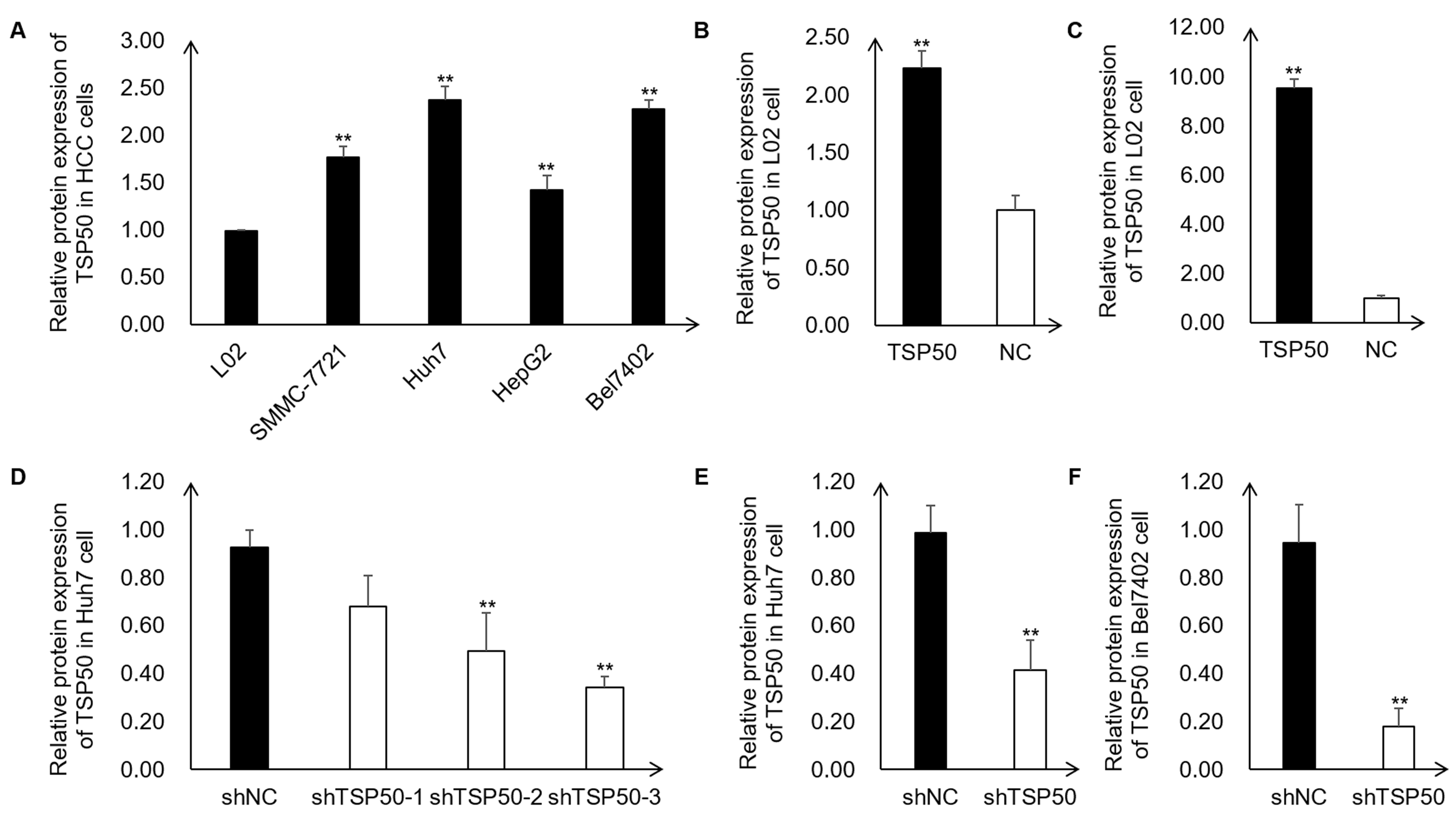

Supplement: Supplementary file 1 — Fig S1 [file CPR-54-e13015-s004.tif]

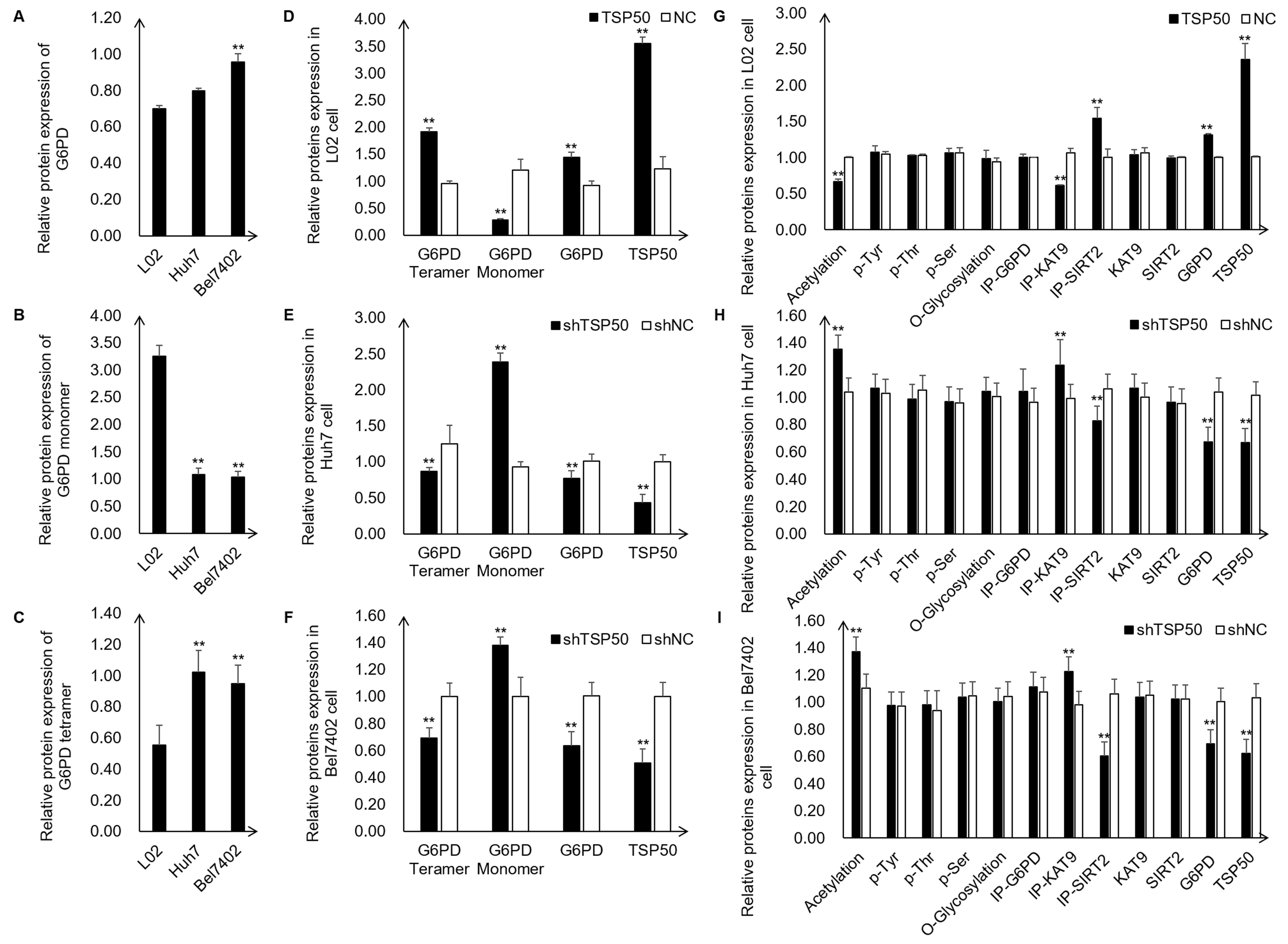

Supplement: Supplementary file 2 — Fig S2 [file CPR-54-e13015-s001.tif]

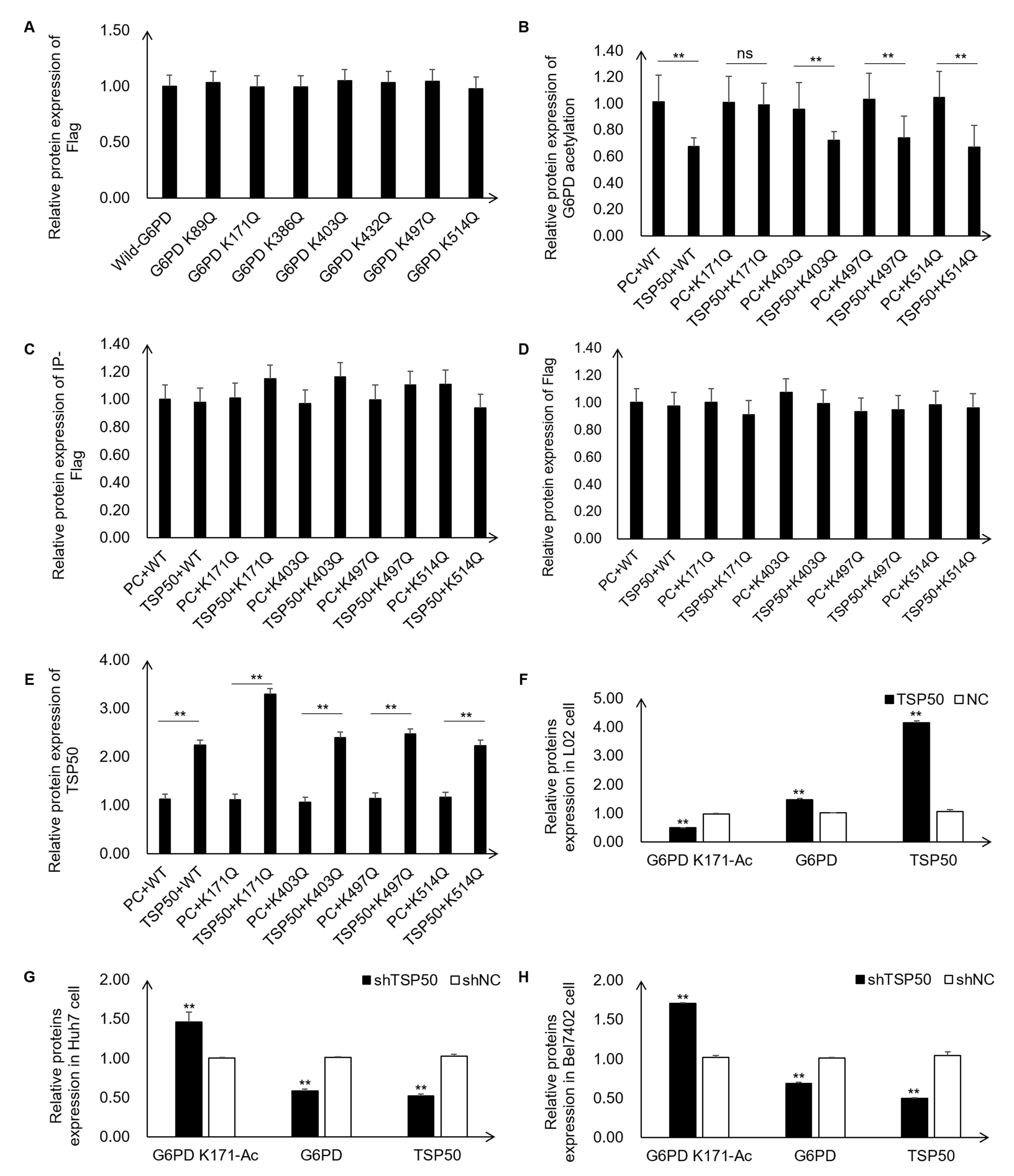

Supplement: Supplementary file 3 — Fig S3 [file CPR-54-e13015-s003.tif]

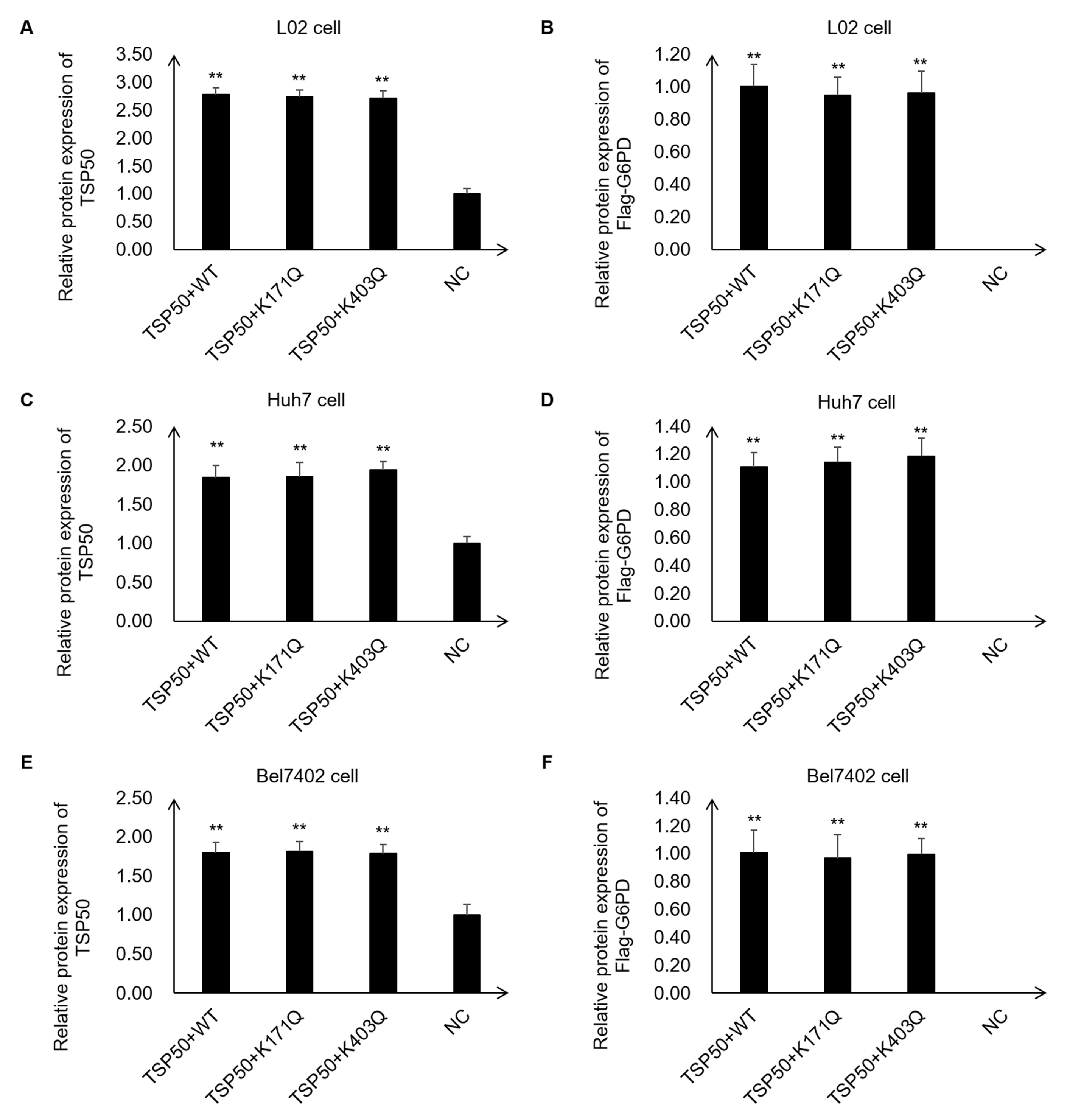

Supplement: Supplementary file 4 — Fig S4 [file CPR-54-e13015-s002.tif]
